# Do you really want to hurt me? The impact of contextual factors on the moderating role of dark leadership in the relationships between learning climate facilitation, employability and turnover intention in the Netherlands and China

**DOI:** 10.3389/fpsyg.2023.1148294

**Published:** 2023-08-03

**Authors:** Omar Habets, Pascale Peters, Beatrice Van der Heijden, Jol Stoffers, Robert Blomme, Shudi Liao

**Affiliations:** ^1^Research Centre for Employability, Zuyd University of Applied Sciences, Sittard, Netherlands; ^2^Center for Strategy, Organization and Leadership, Nyenrode Business Universiteit, Breukelen, Netherlands; ^3^Department of Organisation, Leadership and Management, Inland School of Business and Social Sciences, Lillehammer, Norway; ^4^Institute for Management Research, Radboud University, Nijmegen, Netherlands; ^5^Faculty of Management, Open Universiteit, Heerlen, Netherlands; ^6^Department of Marketing, Innovation and Organisation, Ghent University, Ghent, Belgium; ^7^Business School, Hubei University, Wuhan, China; ^8^Kingston Business School, Kingston University, Kingston upon Thames, United Kingdom; ^9^Research Centre for Education and the Labour Market (ROA), Maastricht University, Maastricht, Netherlands; ^10^Hubei Center for Studies of Human Capital Development Strategy and Policy, Wuhan, China; ^11^Key Research Base of Humanities and Social Science of Hubei Province, Wuhan, China

**Keywords:** learning climate facilitation, dark leadership, employability, turnover intention, cross-national

## Abstract

**Introduction:**

Both the Dutch and Chinese labor markets experience severe shortages of skilled personnel and high turnover rates, being distressing socio-economic factors. At the same time, large cross-cultural studies indicate that these national contexts are highly different from a socio-cultural perspective. When considering issues on employee development and retainment, the public debate opens for negative attributes as dark leadership, wondering if employees accept to be hurt. This study contributes to the employability research and, moreover, it contributes to the call for the ability to contextualize theories and to the convergence/divergence debate. We applied Western theories to investigate possible contextual differences in the relationships between learning climate facilitation and turnover intention, and to investigate whether this relationship is mediated by employability, and whether dark leadership is a possible moderator.

**Methods:**

To test our hypotheses, we collected data from 368 Dutch and 319 Chinese respondents who participate in an executive master’s program, which was analyzed using PLS-Structural Equation Modeling.

**Results:**

Employees in the Netherlands and China were found to interpret our study variables differently. Separate analyses revealed that, in both contexts, learning climate facilitation was both directly and indirectly, via the balance dimension of employability, negatively related to turnover intention. In addition, in the Dutch sample, dark leadership appeared to weaken the relationship between learning climate facilitation and the corporate sense dimension of employability, but the latter did not seem to be a mediator in the relationship with turnover intention. In the Chinese sample, no moderation effects were found.

**Discussion:**

Our results show that both learning climate facilitation and dark leadership are important factors in the development and retainment of personnel and that particularly focusing on ‘balancing group and individual goals’ is important to retain personnel, regardless of national context. The latter may indicate the need for convergence of HR practices. At the same time, however, the different interpretations of the study’s variables may indicate divergence in the meaning of HR concepts. In the discussion section, we elaborate on the study’s implications for HR-researchers and -practitioners in national and global business contexts.

## 1. Introduction

Across the globe, the COVID-19 pandemic has had implications for both people’s daily (e.g., health conditions, distress, and life satisfaction) and working (e.g., working from home) lives (Zhang et al., 2020). In its aftermath, in both Western and Eastern contexts, people are facing socio-economic challenges, such as dealing with substantial labor-market shortages (Cui et al., 2018; CBS, 2019) that cause difficulties for organizations to attract and retain personnel (De Winne et al., 2019). More specifically, organizations experience challenges and instability, which calls for higher levels of employee agility, curiosity, risk mitigation, learning by exploring, and learning by doing, as these characteristics can enhance organizations’ survival chances and competitiveness (Levenson, 2020). In view of this, the importance of a focus on maintaining and enhancing individuals’ employability and facilitating learning opportunities (Buheji and Buheji, 2020) is evident. Employability can be defined as “the continuous fulfilling, acquiring or creating of work through the optimal use of competences” (Van der Heijde and Van der Heijden, 2006, p. 453). Enhancing individuals’ employability implies that organizations need to develop a learning climate that enables and motivates employees to make use of the learning opportunities offered, such that they can indeed increase their individual capabilities and performance (Van der Heijden and Bakker, 2011). Learning climate can be defined as “employees’ perceptions of organizational policies and practices aimed at facilitating, rewarding and supporting employee learning behavior” (Nikolova et al., 2014b, p. 259). This three-dimensional construct refers to facilitation, appreciation, and error avoidance. This study focuses on learning climate facilitation, i.e., organizational efforts to guide, shape and accelerate the learning processes within the organization, since this dimension may help organizations to optimize employees’ process of learning at work (Nikolova et al., 2014b). Particularly, it is found that facilitating employees’ learning opportunities is essential for employee learning and professional development (Marsick and Watkins, 2003; Nikolova et al., 2014a).

Generally, with employability-enhancing activities, organizations may be able to reduce employees’ voluntary turnover intentions (Hom et al., 2012). However, we posit that the relationship between employees’ learning opportunities, employability, and turnover intention may be contingent on how employees perceive and interpret their leaders’ behaviors. Recently, more attention has been paid to dark leadership, in this study operationalized by abusive supervision (Tepper, 2000), as this can affect employees’ motivation to make use of the learning opportunities offered by the organization and, hence, their employability. Dark leadership is defined as “subordinates’ perceptions of the extent to which supervisors engage in the sustained display of hostile verbal and nonverbal behaviors, excluding physical contact” (Tepper, 2000, p. 178). Moreover, research shows that employees who experience dark leadership are more likely to quit their jobs (Tepper et al., 2009). Perhaps, employees who perceive having options to learn and develop themselves, but perceive dark leadership at the same time, may feel they have less opportunities to make use of, or to benefit from their learning options, which may affect their employability and, in turn, may enhance their turnover intention (Acikgoz et al., 2016; Akkermans et al., 2019; Rodrigues et al., 2020).

How employees perceive and respond to Human Resource (HR) practices and leadership style, however, may be contingent on the national context in which they operate of which culture is an important factor. Context refers to the “set of factors surrounding a phenomenon that exert some direct or indirect influence on it” (Whetten, 2009, p. 31). In this vein, Western and Eastern contexts can be characterized by different socio-cultural characteristics; the Netherlands, for example, can be characterized as individualistic, whereas China can be characterized as collectivistic (House et al., 2004; Hofstede Insights, 2022). In view of the call for more context-sensitive organizational research to be able to contextualize theories (Whetten, 2009; Zhu and Warner, 2019; Xiao and Cooke, 2022), this study taps into the convergence-divergence debate as discussed by Cooke et al. (2021). Essentially, this debate started with scholars arguing for the convergence thesis, based on similarities across nations and cultures (*cf.* Harbison and Meyers, 1959), being countered by cross-cultural scholars arguing for the divergence thesis, based on differences in (cultural) norms and values (*cf.* Hofstede, 1991). Addressing the convergence-divergence debate in the Dutch and Chinese contexts gives us reason to question whether employees respond to dark leadership when it comes to the opportunities they take for learning as signaled by their organization’s learning climate and its consequences for employability enhancement and, subsequently, turnover intention. Possibly, in view of the more individualistic context, Dutch employees may notice and respond to dark leadership by altering their investments in their employability and, in turn, their turnover intention. On the contrary, for China, being a country with a more collectivistic character, this reaction of employees may differ due its different contextual characteristics. When considering issues on employee development and retainment, however, such presumed cross-cultural differences are hardly accounted for, neither in HR theory nor in practice, and it can be questioned whether that is an omission in globalizing (labor) markets, as has been discussed in the convergence/divergence debate (*cf.* Cooke et al., 2021).

**Figure 1:**
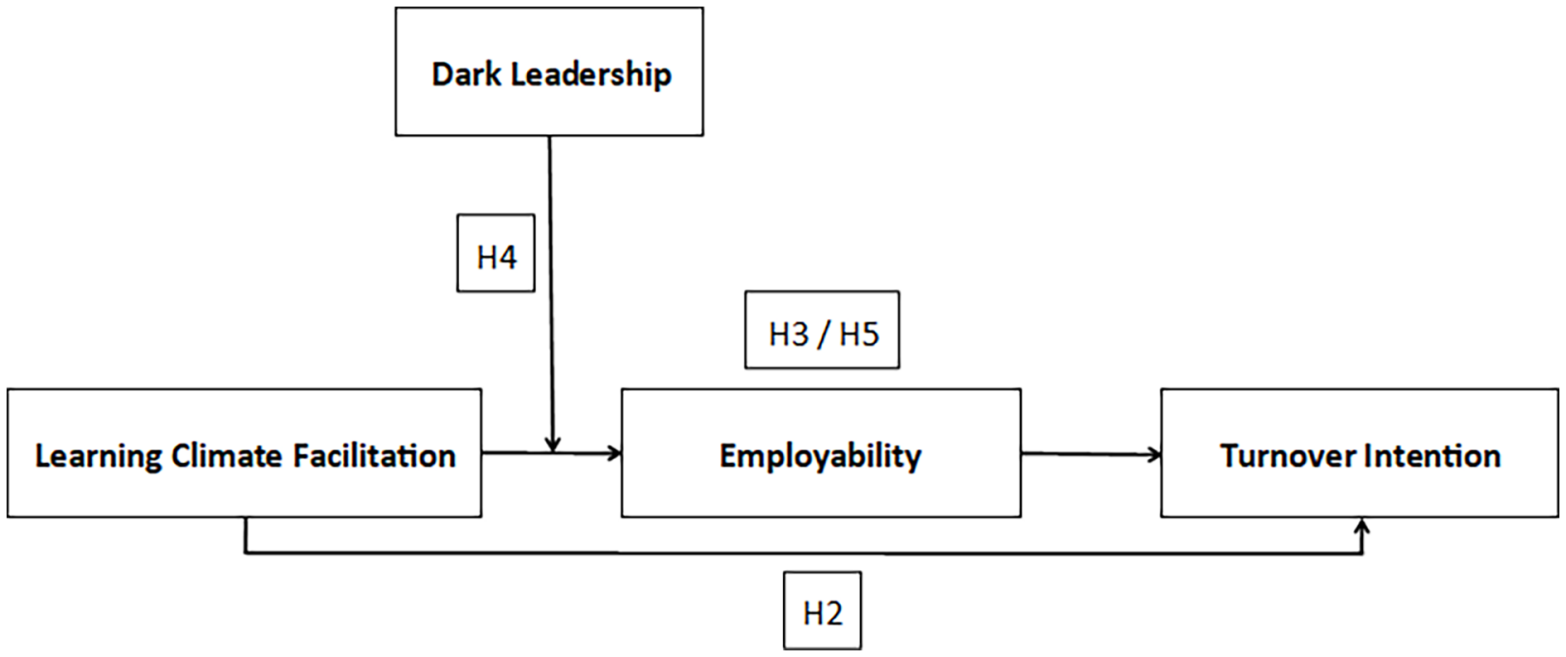
Conceptual model.

This study contributes to the employability research. To the best of our knowledge, no previous research has investigated the relationships between employability and turnover intention, using a competence-based operationalization of employability. In particular, earlier work in the field of turnover intentions and behaviors has conceptualized employability as perceived job chances, both inside and outside one’s current organization (i.e., perceived internal and external employability; De Cuyper et al., 2011a; Nelissen et al., 2017; Baranchenko et al., 2020). Besides, we add to what is already known in the employability literature by investigating the possible moderation effect of dark leadership in the relationship between learning value of the job and employability. Moreover, this study contributes to the call for the ability to contextualize theories and to the convergence/divergence debate. More specifically, this empirical study builds on Western theories and mechanisms to investigate possible contextual differences in the relationships between learning climate facilitation and turnover intention, and whether this relationship is mediated by employability, and whether dark leadership is a possible moderator in this regard. Figure 1 shows the conceptual model that will be tested to address our research question.

## 2. Conceptual framework

### 2.1. Perspectives from the Netherlands and China

Marked by globalization, technological developments, and broader socio-economic, geopolitical, and demographic changes, contemporary complex business contexts require organizations to focus on identifying, attracting, recruiting, developing, and retaining talent (Claus, 2019; Reiche et al., 2019). It has been acknowledged, however, that national context, with their own socio-cultural characteristics, may be an influential factor regarding these focus points. According to Hofstede Insights (2022) and the GLOBE project (House et al., 2004), the Netherlands and China differ dramatically in terms of socio-cultural contextual characteristics. According to Hofstede Insights (2022), the Netherlands is characterized as individualistic with low power distance, high uncertainty avoidance, and high indulgence, whereas China is characterized as collectivistic and as having high power distance, low uncertainty avoidance, and low indulgence. Furthermore, the particular focus on leadership in the GLOBE project shows that leaders in the Netherlands are less autonomous (i.e., the degree to which leaders are independent and individualistic) and less self-protective (i.e., the degree to which leaders focus on ensuring the safety and security of the individual and group through status enhancement and face saving) than leaders in China (House et al., 2004). In empirical research in different national contexts, it is important to note that for meaningfully comparing the mean scores of latent factors, the measurement structures underlying the latent factors used in the research (displayed through a specific set of scale items) should be stable, that is ‘invariant’ (Van de Schoot et al., 2015). Obviously, this is not automatically the case, especially since differences may exist in the prevalence of HR practices (e.g., career development-related ones) in the Netherlands and China (*cf.* Verburg et al., 1999; Van Dierendonck et al., 2016). Also leadership (style) is shown to be deeply rooted in the national contexts’ historical developments (Lin et al., 2018), which makes us believe that leadership behaviors are portrayed differently in the Netherlands and China.

Since socio-cultural characteristics may influence the design and implementation of HR and leadership practices, and their conceptual interpretations (Kim and Wright, 2011), it is crucial to explore the role of socio-cultural characteristics when investigating constructs in different national contexts. Based on the outline given above, we have formulated the following hypothesis:

*H1*: Employees in the Netherlands and China differ regarding their conceptual interpretation of the variables in this study (i.e., learning climate facilitation, dark leadership, employability, and turnover intention).

### 2.2. Learning climate facilitation and turnover intention

Employees’ turnover intentions and their actual quitting behaviors prompt negative organizational outcomes, including financial costs (e.g., recruitment and selection) and loss of highly skilled, talented, and perhaps difficult-to-replace human resources (Lee and Liu, 2007; Hom et al., 2012). High turnover is particularly troublesome for organizations in tight labor markets, given the loss of (firm-specific and general) knowledge and skills, the transfer of that knowledge and skills to a rival organization, and potential deterrence of other employees in the future (Kraimer et al., 2009; Knocke and Schuster, 2017). At the same time, present-day work is changing at an ever-increasing rate and can be constantly restructured in nature (Fugate et al., 2021). This demands of organizations that they have a strong learning climate for their employees that enables them to cope with changing internal and external environments. Even though the construct learning climate by Nikolova et al. (2014b) is three-dimensional (i.e., facilitation, appreciation, and error avoidance), this study focusses on learning climate facilitation only. More specifically, to remain competitive in a fast-changing global economy, an organizational climate that facilitates employees’ workplace learning is essential for an organization to survive (Kyndt et al., 2009; Nikolova et al., 2014b). Allen et al. (2003) already found that perceived organizational support, e.g., the facilitation of a learning climate, can reduce employee turnover. Other research also found that a learning climate reduces negative employee outcomes, such as turnover intention (Egan et al., 2004; Govaerts et al., 2011; Lin and Huang, 2021). This is particularly so since providing a high-quality learning environment signals that organizations value their employees (Egan et al., 2004; Joo, 2010). Hence, we have formulated the following hypothesis:

*H2*: Learning climate facilitation is negatively related with turnover intentions.

### 2.3. The mediating role of employability in the relationship between learning climate facilitation and turnover intention

To survive in contemporary labor markets, protecting and enhancing individuals’ employability is essential (De Vos and Van der Heijden, 2017; Van der Heijden and Spurk, 2019; Stoffers et al., 2020). This, however, is not the responsibility of individual employees alone. Rather, it has been widely acknowledged that organizations should support or facilitate their employees to sustain and enhance their employability (Van Harten et al., 2016; Van der Heijden et al., 2018). Research shows that a learning climate is a precursor to valuable outcomes, such as employees’ learning intentions, positive attitudes toward learning, and participation in learning (Govaerts et al., 2011; Hauer et al., 2012). Indeed, employability can be stimulated by developing an organizational learning climate that promotes employee learning, resulting in the acquisition of new knowledge, skills, and abilities (Nikolova et al., 2016), which provide employees with sufficient opportunities to improve their competencies (Osagie et al., 2018), and hence, their employability (Van der Heijde and Van der Heijden, 2006; Van der Heijden et al., 2018; Crans et al., 2021). This is especially important in an environment characterized by organizational change (Nikolova et al., 2016).

However, there is no consensus on the relationship between employability and turnover intention. This knowledge gap is described in the ‘traditional’ flexibility/commitment paradox (management paradox) (Van der Heijde and Van der Heijden, 2006; De Cuyper and De Witte, 2011). This paradox is defined as “a tension between the contribution of employability to employee well-being and performance, on the one hand, versus its contribution to undesired turnover, on the other hand” (De Cuyper et al., 2011b, p. 1486–1487). In other words, it stresses organizations’ fear of a loss of commitment and external mobility among employees with a high career potential (Van der Heijde and Van der Heijden, 2006) resulting from employability investments. Next to the ‘traditional’ flexibility/commitment paradox, scholars also proposed the notion of the ‘inverted’ flexibility/commitment paradox (Peters and Lam, 2015), which refers to employability aimed at enhancing the organizations’ adaptability, flexibility, effectiveness, and efficiency. In view of this, Human Resource Management (HRM) is aimed at attracting and developing skilled and committed workers who can both easily rotate in their work (i.e., functional flexibility), and who are also focused on external mobility (Peters and Lam, 2015). In this case, the focus is on the idea that HRM’s main goal of their employability policy is not to foster employees’ internal labor market orientation and long-term commitment but rather to enhance both their (short-term) commitment and their flexibility and labor market mobility.

Indeed, empirical research found mixed results in the (indirect) relationship between employability and turnover intention. So far, to the best of our knowledge, all research dealing with the relationship between employability and turnover intention incorporated measures of employability conceptualizing perceived job chances at the internal and/or external labor markets. For example, Hom et al. (1992) found a positive association between employability and turnover intention and while testing the employability paradox, Nelissen et al. (2017) also found that employability relates positively to turnover intention, however, only in terms of upward career development.

On the other hand, De Cuyper et al. (2011a) found no direct relationship between employability and turnover intention, and Akkermans et al. (2019) reported that perceived investments in HR practices relate positively with organizational commitment. Recently, Rodrigues et al. (2020) found that when an organization invests in employees’ career development (and in case the employee is receptive), it has a positive impact on their intention to stay with the employer, and, moreover, they found a negative association between employability and turnover intention.

Building on these mixed results, we strive to shed more light on the phenomenon by introducing a competence-based operationalization of employability (Van der Heijde and Van der Heijden, 2006; Van der Heijden et al., 2018) that mediates the negative relationship between learning climate facilitation and turnover intention. Hence, we have formulated the following hypothesis:

*H3*: Employability mediates the negative relationship between learning climate facilitation and turnover intention.

### 2.4. The moderating role of dark leadership in the relationship between facilitating learning climate and employability

Scholarly work in the field of leadership focuses mainly on constructive leadership styles since leadership behavior that supports and reinforces learning can enhance employability (Stoffers et al., 2019). However, researchers in other fields have also investigated the phenomenon of dark leadership. This has not been done without reason, since research showed that destructive leadership is an enduring problem for organizations due to its adverse and expensive effects on turnover (Tepper et al., 2006). The recent literature review by Mackey et al. (2021) gave an overview of definitions for various styles of destructive leadership. Abusive supervision (Tepper, 2000) has been especially influential in the literature (Mackey et al., 2021). The recent systematic reviews on abusive supervision by Fischer et al. (2021) and Goute et al. (2021) show that Tepper (2000) definition of abusive supervision is widely used in empirical research. The reviews referred to above presented us with a rather uniform picture: abusive supervision is ‘bad news’ (Fischer et al., 2021). Furthermore, another recent meta-analytic review by Landay et al. (2019) indicated a concern regarding the potential prevalence of individuals with psychopathic tendencies in corporate leadership positions and the negative effects they may have on both individuals and their organizations in practice. They found a negative association for psychopathic tendencies and leadership effectiveness amongst others towards employees. The meta-analysis by Schyns and Schilling (2013) also demonstrated negative correlations between dark leadership, on the one hand, and positive follower outcomes and behaviors (e.g., attitudes toward a leader, wellbeing, and individual performance), on the other hand, and, similarly, positive correlations with negative outcomes (e.g., turnover intentions, resistance toward a leader, and counterproductive work behaviors).

To the best of our knowledge, up until now, the phenomenon of dark leadership has not been investigated in relation to workers’ employability. Incorporating the possible role of dark leadership as a moderator in the relationship between facilitating learning climate and employability is crucial given the dual responsibility for employability enhancement, that is both the employee and the supervisor need to invest in these (Van der Heijden, 2005).

Possibly, employees who perceive having options to learn and to develop themselves but who perceive dark leadership at the same time, may feel less motivated due to reduced self-efficacy, and, as such, they perceive to have fewer opportunities to make use of the learning options offered by the organization. The latter may affect their employability and, ultimately, may have consequences for their turnover intention (Acikgoz et al., 2016; Akkermans et al., 2019; Le Blanc et al., 2019; Rodrigues et al., 2020). In other words, in a context in which employees experience dark leadership, employees may be less motivated and more hindered to take advantage of the learning options offered, which may affect their employability, and, indirectly, their turnover intention. Based on the outline given above, we have formulated the following hypotheses:

*H4*: Dark leadership moderates the positive relationship between learning climate facilitation and employability, such that this relationship is weaker for employees who experience a higher degree of dark leadership.

*H5*: The interaction effect between dark leadership and learning climate facilitation has an indirect effect through employability on turnover intention.

## 3. Methods

### 3.1. Participants and procedures

To test our hypotheses, we used a quantitative approach with a cross-sectional research design. To comply with the need to conduct more research with higher levels of contextual analysis, we gathered data from the Netherlands and China, as they experience similar socio-economic challenges, such as labor shortages and the COVID-19 pandemic but, at the same time, have different national contexts (Cui et al., 2018; CBS, 2019). China is particularly interesting to incorporate in our study as the opening-up policy and its reforms in 1978 have led to remarkable economic performance, primarily because of its abundance of cheap labor (Cui et al., 2018). Correspondingly, the Chinese labor market has experienced high-speed developments over the past decennia. More specifically, over the last three decades, HRM has emerged as a discipline and research field of interest for (Chinese) scholars (Cooke et al., 2021) making human capital a highly interesting topic to be studied empirically.

In both national contexts, respondents were approached through the research team’s personal contacts (i.e., convenience sampling). They were employees at middle and higher management levels, with some years of work experience and were recruited via several part-time master’s programs. In return for participating in the research, all respondents received a personalized employability report of their own employability scores set off against a benchmark. The respondents could use this in their personal development component of their master’s program. The final sample comprised 382 Dutch and 319 Chinese respondents. In the Dutch sample 46% were male and 54% female, and the average age was 47.34 years (SD = 10.13). Furthermore, 79% of the respondents had a fixed contract, 12% a temporary contract, and 9% were self-employed. In the Chinese sample 59% were male and 41% female with an average age of 37.24 (SD = 4.58). In this Chinese sample, 85% had a fixed contract, 9% a temporary contract, and 6% were self-employed.

### 3.2. Measures

Learning climate facilitation was measured using a validated scale (Nikolova et al., 2014a,b), comprising three items. A sample item was “My organization provides appealing learning opportunities.” Responses were reported using a Likert-type scale that ranged from 1 (not applicable at all) to 5 (fully applicable).

Dark leadership was measured using the abusive supervision scale (Tepper, 2000). Respondents completed Tepper (2000) 15-item measure of abusive leadership. A sample item was: “My boss tells me my thoughts or feelings are stupid.” The items were scored using a Likert-type scale that ranged from 1 (strongly disagree) to 7 (strongly agree).

Employability was measured using the thoroughly validated 22-item short form version (Van der Heijden et al., 2018) of the 47-item employability five-factor instrument (Van der Heijde and Van der Heijden, 2006). Tests of the instrument’s reliability and validity, including assessments of convergent, discriminant, and predictive validity (for career success), have yielded satisfactory psychometric results (Van der Heijden et al., 2018). The instrument comprises the following dimensions: occupational expertise (5 items); anticipation and optimization (4 items); personal flexibility (5 items); corporate sense (4 items); and balance (4 items). Sample items for employability include: “I consider myself competent to provide information on my work in a way that is comprehensible” (occupational expertise); “I consciously devote attention to applying my newly acquired knowledge and skills” (anticipation and optimization); “I adapt to developments within my organization” (personal flexibility); “I share my experience and knowledge with others” (corporate sense); and “I achieve a balance in alternating between reaching my own career goals and supporting my colleagues” (balance). All items were scored using a Likert-type scale that ranged from 1 (not at all/never) to 6 (considerable degree/very often), depending on an item’s wording.

Turnover intention was measured using a 3-item scale from Camman et al. (1979). A sample item was “I often think of leaving the organization.” Items were scored using a Likert-type scale that ranged from 1 (totally disagree) to 5 (totally agree).

### 3.3. Analyses

We conducted PLS-SEM, using the SmartPLS version 4.0.8.5 (Ringle et al., 2022). For the partial least square algorithm, we used the path weighting scheme, and we set the maximum number of iterations at 300. We used 10^-5 as our stop criterion and a uniform value of 1 as the initial value for each of the outer weights (Henseler, 2010). The sample size was acceptable using Barclay et al. (1995) rule of thumb, suggesting the use of ten times the maximum number of paths aiming at any construct in the outer and inner models. Items were based on 5-point, 6-point and 7-point Likert-scales and could be interpreted as continuous variables, thus following the fundamental OLS principles.

## 4. Results

### 4.1. Model characteristics

For the outer model evaluation, we first examined reliability. All the scales appeared to be reliable (Nunnally, 1978) and no item had to be removed (see Table 1 for all details). Second, we checked for convergent validity using Fornell and Larcker’s criterion of an Average Variance Extracted (AVE) for each construct above the 0.5 benchmark (Fornell and Larcker, 1981). In this respect, we also did not have to remove any items to have sufficient convergent validity (see Table 1).

**Table 1:** Overview reliability and construct validity scores of the constructs.

	*N*	Theoretical range	Actual range	Mean	SD	Cronbach alfa	Ave.
Learning climate facilitation	687	1.00–5.00	1.00–5.00	3.24	0.90	0.85	0.77
Dark leadership	687	1.00–7.00	1.00–7.00	2.12	1.09	0.95	0.62
Occupational expertise	687	1.00–7.00	1.40–6.00	4.75	0.61	0.8	0.54
Anticipation and optimization	687	1.00–7.00	2.00–6.00	4.11	0.73	0.74	0.55
Personal flexibility	687	1.00–7.00	1.00–6.00	4.6	0.6	0.8	0.55
Corporate sense	687	1.00–7.00	1.25–6.00	4.35	0.78	0.75	0.57
Balance	687	1.00–7.00	1.50–6.00	3.88	0.77	0.75	0.55
Turnover intention	687	1.00–5.00	1.00–5.00	2.93	0.96	0.8	0.69

Finally, we checked for discriminant validity, comparing the AVEs of the constructs with the inter-construct correlations determining whether each latent variable shared greater variance with its own measurement variables or with other constructs (Fornell and Larcker, 1981; Chin, 1998). We compared the square root of the AVE for each construct with the correlations with all other constructs in the model (see Table 2). A correlation between constructs exceeding the square roots of their AVEs indicates that they may not be sufficiently discriminable (see Table 2 for all details). For each construct, we found that the absolute correlations did not exceed the square roots of the AVEs. As such, there was support for sufficient reliability and validity of all measurements in our research model.

**Table 2:** Correlation table, numbers shown in boldface denote the square root of the average variance extracted.

	1	2	3	4	5	6	7	8
Learning climate facilitation (1)	**0.89**							
Dark leadership (2)	−0.38**	**0.7**						
Occupational expertise (3)	0.17	−0.13**	**0.73**					
Anticipation and optimization (4)	0.28**	−0.14**	0.40**	**0.74**				
Personal flexibility (5)	0.32**	−0.30**	0.50**	0.48**	**0.74**			
Corporate sense (6)	0.30**	−0.24**	0.38**	0.60**	0.52**	**0.75**		
Balance (7)	0.41**	−0.36**	0.22**	0.25**	0.34**	0.30**	**0.74**	
Turnover intention (8)	−0.52**	0.39**	−0.06	−0.22**	−0.20**	−0.25**	−0.41**	**0.83**

Significance correlations: ^**^p < 0.01.

### 4.2. Common-method variance

As this research was conducted using a self-administered survey method, we tested for Common-Method Variance (CMV) to have evidence that there is no systematic bias which might have influenced the collected data (Podsakoff et al., 2003). We used a two-step approach. First, following Podsakoff and Organ (1986), we used Harman (1976) one-factor test. Following this approach, all principal constructs were entered into one principal component factor analysis. Using SPSS Software (SPSS version 27 for MAC OS), the one-factor extraction and non-rotation method was applied. Results showed that only one factor emerged, explaining less than 50 percent of the variance (25.20%), which gives a first indication of no common-method variance. Second, we used Bagozzi’s method (Bagozzi et al., 1991) which stresses that CMV occurs when the highest correlation between constructs is more than 0.9. As shown in Table 2, the highest correlation between constructs is 0.60 (correlation between Anticipation and Corporate Sense). Therefore, it appears that there is no CMV in the collected data.

### 4.3. Differences between the Dutch and Chinese sample

To find out whether the group of Dutch and Chinese respondents interpreted the study’s core variables in a similar manner, we used the Measurement Invariance of Composite Models (MICOM) procedure (*cf.* Hair et al., 2017), which is considered a proper avenue to a group’s uniqueness when exploring significant differences between two groups (Sarstedt et al., 2011; Henseler et al., 2016). The first step of the MICOM procedure is to test for configural invariance to examine whether each common factor was associated with identical item sets across the two distinguished groups. Configural invariance means that a latent variable, which has been specified equally, emerges as a unidimensional entity across the two groups (Henseler et al., 2016). As shown in Table 1, and as discussed in the previous section, all constructs showed sufficient reliability and validity for both groups and were used as an input for the next step.

As a second step, the compositional invariance was tested. The results indicate whether the latent variables are formed differently in the two groups (Henseler et al., 2016). In this analysis, the correlation (*r*) between, on the one hand, the composite scores using the weights obtained from the first group and, on the other hand, the composite scores using the weights obtained from the second group, were compared using a permutation test (Henseler et al., 2016). Henseler et al. (2016) proposed that the correlation *r* should be equal to one. If the correlation *c* diverges significantly from 1, no support is found for compositional invariance. To statistically test for compositional invariance, we proposed a two-step procedure. The second step comprises q permutation test over the correlation *c* (Chin and Dibbern, 2010). The outcomes of the second step in the permutation test yielded insufficient support for compositional invariance (Henseler et al., 2016), given that for multiple variables (i.e., corporate sense and dark leadership) the correlations *c* between the two distinguished groups were significantly different. As such, no support was found for compositional invariance (see Table 3). Furthermore, in step 3 (*cf.* Hair et al., 2017), in which the differences between the equal means (see Table 4) and equal variances (see Table 5) are calculated, also significant differences for most of the constructs were found.

**Table 3:** Step 2 in the MICOM procedure.

	Original correlation	Correlation permutation mean	5.0%	Permutation *p* value
Learning climate facilitation	1	1	1	0.66
Dark leadership	1	1	1	0.04
Occupational expertise	0.89	0.94	0.84	0.11
Anticipation and optimization	0.97	0.99	0.97	0.09
Personal flexibility	0.99	0.99	0.98	0.14
Corporate sense	0.98	0.99	0.98	0.05
Balance	1	1	0.99	0.43
Turnover intention	1	1	1	0.80

**Table 4:** Step 3a in the MICOM procedure (mean).

	Original difference	Permutation mean difference	2.5%	97.5%	Permutation *p* value
Learning climate facilitation	−0.34	0.00	−0.14	0.16	0.00
Dark leadership	0.60	0.00	−0.16	0.15	0.00
Occupational expertise	−0.07	0.00	−0.15	0.15	0.36
Anticipation and optimization	−0.35	−0.01	−0.17	0.14	0.00
Personal flexibility	−0.64	0.00	−0.16	0.15	0.00
Corporate sense	−0.46	0.00	−0.14	0.14	0.00
Balance	−0.28	0.00	−0.16	0.16	0.00
Turnover intention	0.06	0.00	−0.14	0.16	0.46

**Table 5:** Step3b MICOM procedure (variances).

	Original difference	Permutation mean difference	2.5%	97.5%	Permutation *p* value
Learning climate facilitation	−0.05	0.00	−0.19	0.18	0.58
Dark leadership	−0.04	0.00	−0.27	0.26	0.75
Occupational expertise	0.13	0.00	−0.28	0.30	0.38
Anticipation and optimization	−0.26	0.00	−0.21	0.22	0.02
Personal flexibility	0.08	0.00	−0.28	0.27	0.59
Corporate sense	−0.28	0.00	−0.20	0.19	0.04
Balance	0.23	0.00	−0.24	0.21	0.05
Turnover intention	−0.34	0.00	−0.19	0.17	0.00

The MICOM analysis demonstrated insufficient measurement invariance for both groups, which indicates that the group of Dutch respondents interpreted the measures in a conceptually different way compared to their Chinese counterparts. This outcome provides support for Hypothesis 1 [Employees in the Netherlands and China differ regarding their conceptual interpretation of the variables in this study (i.e., learning climate facilitation, dark leadership, employability, and turnover intention)], as the constructs of learning climate facilitation, dark leadership, employability, and turnover intention demonstrated significantly different interpretations across the two national contexts.

### 4.4. Model estimations

Regarding the inner model evaluation and estimates, we analyzed the path coefficients by using bootstrap t-statistics, using 5,000 subsamples, with a bias-corrected bootstrap, testing for a two-tailed significance of 95% (Anderson and Gerbing, 1988). The model showed a good fit to our data: the Standardized Root Mean Square Residual (SRMR) was 0.06, which is in line with Hu and Bentler (1998) criterion of a lower value than 0.08. As the MICOM procedure demonstrated that the groups were different, we tested Hypotheses 2, 3, 4 and 5 for the Dutch and Chinese groups of respondents separately.

To test the hypotheses, we used a two-step approach. First, we calculated the direct effects for the paths in the model (see Tables 6a, 7a). Second, we calculated the indirect effects for the paths in the model (see Tables 6b, 7b). Last, we tested the predicting power using f^2^ effect size of Cohen (1988) in the direct effects to indicate whether each construct had a weak, average, or strong effect on each of the related constructs (see Tables 6a, 7a).

**Table 6a:** Direct effects Dutch respondents.

	*γ*	Standard deviation	T statistics	*f*^2^ values	*p* values
Learning climate facilitation - > Occupational expertise	0.16	0.09	1.79	0.02	0.07
Learning climate facilitation - > Anticipation and optimization	0.25	0.05	4.62	0.06	0.00
Learning climate facilitation - > Personal flexibility	0.19	0.05	3.48	0.03	0.00
Learning climate facilitation - > Corporate sense	0.21	0.06	3.68	0.04	0.00
Learning climate facilitation - > Balance	0.28	0.05	5.77	0.08	0.00
Learning climate facilitation - > Turnover intention	−0.43	0.04	9.80	0.25	0.00
Dark leadership × Learning climate facilitation - > Occupational expertise	0.07	0.08	0.85	0.00	0.40
Dark leadership × Learning climate facilitation - > Anticipation and optimization	0.01	0.05	0.24	0.00	0.81
Dark leadership × Learning climate Facilitation - > Personal flexibility	0.00	0.05	0.04	0.01	0.97
Dark leadership × Learning climate facilitation - > Corporate sense	−0.14	0.06	2.46	0.01	0.01
Dark leadership × Learning climate facilitation - > Balance	0.06	0.06	1.03	0.03	0.30
Dark leadership - > Occupational expertise	−0.06	0.08	0.69	0.00	0.49
Dark leadership - > Anticipation and optimization	−0.02	0.07	0.23	0.00	0.82
Dark leadership - > Personal flexibility	−0.14	0.07	2.11	0.01	0.04
Dark leadership - > Corporate sense	−0.13	0.07	1.90	0.01	0.06
Dark leadership - > Balance	−0.19	0.07	2.98	0.03	0.00
Occupational expertise - > Turnover intention	0.10	0.07	1.52	0.01	0.13
Anticipation and optimization - > Turnover intention	−0.10	0.05	1.92	0.01	0.06
Personal flexibility - > Turnover intention	0.04	0.06	0.80	0.00	0.42
Corporate sense - > Turnover intention	−0.07	0.05	1.45	0.00	0.15
Balance - > Turnover intention	−0.26	0.05	5.03	0.09	0.00

**Table 6b:** Indirect effects Dutch sample.

	*γ*	Standard deviation	T statistics	*p* values
Learning climate facilitation - > Occupational expertise - > Turnover intention	0.02	0.01	1.09	0.28
Learning climate facilitation - > Anticipation and optimization - > Turnover intention	−0.03	0.01	1.79	0.07
Learning climate facilitation - > Personal flexibility - > Turnover intention	0.01	0.01	0.73	0.46
Learning climate facilitation - > Corporate sense - > Turnover intention	−0.02	0.01	1.29	0.20
Learning climate facilitation - > Balance - > Turnover intention	−0.07	0.02	3.77	0.00
Dark leadership × Learning climate facilitation - > Occupational expertise - > Turnover intention	0.01	0.01	0.74	0.46
Dark leadership × Learning climate facilitation - > Anticipation and optimization - > Turnover intention	0.00	0.01	0.22	0.83
Dark leadership × Learning climate facilitation - > Personal flexibility - > Turnover intention	0.00	0.00	0.02	0.98
Dark leadership × Learning climate facilitation - > Corporate sense - > Turnover intention	0.01	0.01	1.19	0.24
Dark leadership × Learning climate facilitation - > Balance - > Turnover intention	−0.02	0.02	1.00	0.32
Dark leadership - > Occupational expertise - > Turnover intention	−0.01	0.01	0.54	0.59
Dark leadership - > Anticipation and optimization - > Turnover intention	0.00	0.01	0.21	0.84
Dark leadership - > Personal flexibility - > Turnover intention	−0.01	0.01	0.70	0.49
Dark leadership - > Corporate sense - > Turnover intention	0.01	0.01	0.95	0.34
Dark leadership - > Balance - > Turnover intention	0.05	0.02	2.60	0.01

**Table 7a:** Direct effects Chinese respondents.

	*γ*	Standard deviation	T statistics	*f*^2^ values	*p* values
Learning climate facilitation - > Occupational expertise	0.14	0.08	1.69	0.02	0.09
Learning climate facilitation - > Anticipation and optimization	0.31	0.06	5.14	0.10	0.00
Learning climate facilitation - > Personal flexibility	0.32	0.06	5.53	0.11	0.00
Learning climate facilitation - > Corporate sense	0.29	0.06	5.32	0.09	0.00
Learning climate facilitation - > Balance	0.35	0.05	6.91	0.15	0.00
Learning climate facilitation - > Turnover intention	−0.36	0.05	6.93	0.15	0.00
Dark leadership × Learning climate facilitation - > Occupational expertise	−0.10	0.07	1.54	0.01	0.13
Dark leadership × Learning climate facilitation - > Anticipation and optimization	−0.08	0.06	1.25	0.01	0.21
Dark leadership × Learning climate facilitation - > Personal flexibility	−0.10	0.06	1.55	0.01	0.12
Dark leadership × Learning climate facilitation - > Corporate sense	−0.06	0.06	1.03	0.00	0.30
Dark leadership × Learning climate facilitation - > Balance	−0.05	0.06	0.95	0.00	0.34
Dark leadership - > Occupational expertise	−0.14	0.08	1.62	0.02	0.11
Dark leadership - > Anticipation and optimization	0.01	0.08	0.13	0.00	0.90
Dark leadership - > Personal flexibility	−0.20	0.07	2.99	0.04	0.00
Dark leadership - > Corporate sense	−0.19	0.07	2.72	0.04	0.01
Dark leadership - > Balance	−0.26	0.06	4.59	0.07	0.00
Occupational expertise - > Turnover intention	0.12	0.06	1.90	0.01	0.06
Anticipation and optimization - > Turnover intention	0.02	0.07	0.30	0.00	0.76
Personal flexibility - > Turnover intention	−0.06	0.06	0.88	0.00	0.38
Corporate sense - > Turnover intention	−0.13	0.07	1.98	0.01	0.05
Balance - > Turnover intention	−0.23	0.06	4.22	0.06	0.00

**Table 7b:** Indirect effects Chinese respondents.

	*γ*	Standard deviation	T statistics	*p* values
Learning climate facilitation - > Occupational expertise - > Turnover intention	0.02	0.01	1.17	0.24
Learning climate facilitation - > Anticipation and optimization - > Turnover intention	0.01	0.02	0.30	0.77
Learning climate facilitation - > Personal flexibility - > Turnover intention	−0.02	0.02	0.85	0.40
Learning climate facilitation - > Corporate sense - > Turnover intention	−0.04	0.02	1.84	0.07
Learning climate facilitation - > Balance - > Turnover intention	−0.08	0.02	3.92	0.00
Dark leadership × Learning climate facilitation - > Occupational expertise - > Turnover intention	−0.01	0.01	1.11	0.27
Dark leadership × Learning climate facilitation - > Anticipation and Optimization - > Turnover intention	0.00	0.01	0.23	0.82
Dark leadership × Learning climate facilitation - > Personal flexibility - > Turnover intention	0.01	0.01	0.70	0.49
Dark leadership × Learning climate facilitation - > Corporate sense - > Turnover intention	0.01	0.01	0.83	0.41
Dark leadership × Learning climate facilitation - > Balance - > Turnover intention	0.01	0.01	0.89	0.37
Dark leadership - > Occupational expertise - > Turnover intention	−0.02	0.01	1.33	0.18
Dark leadership - > Anticipation and optimization - > Turnover intention	0.00	0.01	0.04	0.97
Dark leadership - > Personal flexibility - > Turnover intention	0.01	0.01	0.78	0.44
Dark leadership - > Corporate sense - > Turnover intention	0.03	0.02	1.38	0.17
Dark leadership - > Balance - > Turnover intention	0.06	0.02	2.76	0.01

Hypothesis 2 (Learning climate facilitation is negatively related with turnover intentions) was supported for both groups with our data. For the Dutch respondents, learning climate facilitation had a significant negative relationship with turnover intention (*γ* = −0.43, *p* < 0.00, *R^2^* = 0.37) with a strong predictive power (*f^2^* = 0.25). For the Chinese respondents, learning climate also facilitation had a significant negative relationship with turnover intention (*γ* = −0.36, *p* < 0.00, *R^2^* = 0.33) with an average predictive power (*f^2^* = 0.15).

Hypothesis 3 (Employability mediates the negative relationship between learning climate facilitation and turnover intention) was partly supported for both groups. For the Dutch respondents, learning climate facilitation had a significant negative relationship with turnover intention via balance (*γ* = −0.07, *p* < 0.000, *R^2^* = 0.37). For the Chinese respondents, learning climate facilitation also had a significant negative relationship with turnover intention via balance (*γ* = −0.08, *p* < 0.00, *R^2^* = 0.33). Indirect paths with the other components of employability as mediator demonstrated to be non-significant.

Hypothesis 4 (Dark leadership moderates the positive relationship between learning climate facilitation and employability, such that this relationship is weaker for employees who experience a higher degree of dark leadership) was partially supported for the Dutch respondents; a significant moderation effect between dark leadership and learning climate facilitation was found on corporate sense (*γ* = −0.14, *p* < 0.01, *R^2^* = 0.37). However, for the Chinese respondents, no significant relationship was found for the moderation between dark leadership and learning climate facilitation on each of the components of employability.

Hypothesis 5 (The interaction effect between dark leadership and learning climate facilitation has an indirect effect through employability on turnover intention) was neither supported for the Dutch nor for the Chinese group of respondents, as no significant indirect relationship was found of the moderation between dark leadership and learning climate facilitation and turnover intention via each of the components of employability.

## 5. Discussion and conclusion

In view of the call for the ability to contextualize theories (Whetten, 2009) and to contribute to the convergence/divergence debate, this empirical study applied Western theories to investigate possible contextual differences in the relationships between learning climate facilitation and turnover intention, and to examine whether this relationship is mediated by employability, and the possible role of dark leadership as a moderator in our research model. To test our hypotheses, we collected data from 319 Chinese and 368 Dutch respondents, which were analyzed using PLS-Structural Equation Modeling. Below, we summarize our results and reflect on them in the light of our theoretical lens. We conclude by discussing the study’s implications for HR researchers and - practitioners in national and global business contexts.

In line with our expectations, employees in the Netherlands and China were shown to interpret our study variables differently. More specifically, our results indicated that learning climate facilitation, dark leadership, employability, and turnover intention are significantly differently interpreted across the two national contexts studied. More generally, this would argue for the divergence thesis as brought forward by cross-cultural scholars when it comes down to the interpretation of our Western concepts related to HRM and leadership in China.

Our separate analyses revealed a significant negative relationship between learning climate facilitation and turnover intention in both the Netherlands and China. Although not directly comparable with each other (see Hypothesis 1), we found a negative relationship between the two constructs in both contexts. Since employees in both contexts can be expected to have lower turnover intentions when they perceive a learning climate facilitation within their organization, this supports the convergence thesis. This finding is also in line with recent research by Lin and Huang (2021) that a learning climate reduces turnover intention. Although this was not hypothesized, in both samples, we found that learning climate facilitation also had a positive direct relationship with employability (anticipation and optimization, personal flexibility, corporate sense, and balance) (see Tables 6a, 7a). This means that in both the Netherlands and China, efforts of an organization to facilitate a learning climate are beneficial to the employability development of their employees.

Another notable result is that, in both contexts, learning climate facilitation had a significant negative relationship with turnover intention via the competence based employability dimension of balance. In this empirical work, balance refers to the capability to align one’s current job versus career goals, employers’ versus employees’ interests, and employees’ opposing work, career, and private interests (Van der Heijde and Van der Heijden, 2006). Considering the lack of consensus on the relationship between employability and turnover intention in empirical research, this study shows, in line with the ‘inverted’ flexibility/commitment paradox (Peters and Lam, 2015), that employees would have lower intentions to leave the organization when they experience the balance between organizational goals and individual goals to be right. A possible explanation for this outcome may be that employees (in our sample those who participate in an executive master’s program) who believe to have more learning opportunities, experience a higher form of balance (in this case by experiencing that their employer’s and their own interests are taken care of), resulting into lower turnover intentions. We find a similar relationship between both contexts, which also supports the convergence thesis. Interestingly, this is in line with recent research (Burmeister et al., 2018) that also found that the effects of HR practices do not significantly differ when comparing collectivist and individualist countries.

Furthermore, in this empirical study it was found that in the Dutch context, dark leadership moderates the relationship between learning climate facilitation and the employability dimension of corporate sense, meaning that the positive relationship between learning climate facilitation and corporate sense [e.g., networking skills, actual participation in different types of working groups, and sharing responsibilities, expertise, successes *et cetera* (Van der Heijde and Van der Heijden, 2006)] is weakened by the experience of dark leadership. In other words, the positive contribution of learning climate facilitation to corporate sense is hindered by dark leadership.

In contrast to the Dutch respondents, there is no significant result in the Chinese context for a moderation of dark leadership in our model. This supports the divergence thesis, stressing contextual differences in HR practices and leadership style. It may well be that the historical developments of leadership (Lin et al., 2018) and employees in the Netherlands and China make for actual differences in current times. Where Chinese leaders are more autonomous and more self-protective compared to leaders in the Netherlands and Chinese employees are more collectivistic (House et al., 2004), it may well be that Chinese employees are not affected by dark leadership and do not experience it as a hinderance in the relationship between learning climate facilitation and employability.

Finally, no significant relationship was found for the moderation of dark leadership in the relationship between learning climate facilitation and turnover intention running via the employability dimensions. In contrast to our expectations, experienced dark leadership does not indirectly affect employees’ turnover intention via employability in either the Dutch or the Chinese context. Yet, we did find a direct negative relationship of dark leadership with employability in the Netherlands (personal flexibility and balance) and China (personal flexibility, corporate sense, and balance), implying that employees who experience dark leadership perceive lower levels on selected dimensions of employability. Overall, our finding is in line with the rather uniform picture presented by Fischer et al. (2021) indicating that abusive supervision is ‘bad news’.

Based on our study, on the one hand, it can be concluded that both learning climate facilitation and dark leadership are important factors in the development and retainment of personnel and that particularly focusing on ‘balancing employer and individual goals’ can be considered as particularly important to retain personnel, regardless of the national context being the Netherlands or China. To some extent, this may indicate the need for convergence of HR practices regarding learning climate facilitation.

On the other hand, however, the different interpretations of our study’s variables may indicate divergence in the meaning of HR concepts in the two national contexts studied. In line with the call for more context-sensitive organizational research to be able to contextualize theories (Whetten, 2009; Zhu and Warner, 2019; Xiao and Cooke, 2022), our study contributes to the understanding of contextual differences, while using Western theories. More specifically, our study contributes to the extant literature on learning climate facilitation, dark leadership, employability, and turnover intention, by providing insights on contextual similarities and differences.

### 5.1. Limitations and future research

Like other studies, this study also has some limitations. First, data was collected using surveys, and thus response set consistencies and common-method biases may be a potential concern (Doty and Glick, 1998). However, in the study’s design, we reduced common-method variance by using brief questionnaires and a combination of response formats and labeling (Podsakoff et al., 2012).

Second, this study exclusively used self-ratings, and thus more research is needed to assess how this might have affected results. Future research can also use a qualitative interpretation to increase the understanding of the results.

Third, concerning measures of our mediation variable, extant studies suggested that other-ratings of employability are less differentiated and result in less variance between the employability sub-dimensions distinguished (Van der Heijden et al., 2016). Self-ratings of employability were thus justified to obtain nuanced findings. However, future research could use multi-source data to further enhance our understanding of the influence of learning climate facilitation on employees’ turnover intentions.

Fourth, related to the previous limitation, a multi-wave approach would have provided more data on the stability and change of variables in the model, and longitudinal, cross-lagged relationships compared with the current cross-sectional design (Boker and Nesselroade, 2002). Future studies could assess reciprocal relationships, such as cross-lagged effects between antecedents and outcomes (Kwok and Fang, 2021). For example, employees with higher turnover intention who focus on external collaboration and networks might become more employable over time as well, thereby suggesting positive reciprocal relationships between the study’s variables.

Finally, our sample was limited to employees who were allowed by their employer to participate in an executive master’s program limited to two countries, i.e., the Netherlands and China. This may have diminished the generalizability of our study, as it is likely that employees who at the same time are participating in such an educational program, may have a more positive impression of their learning opportunities in comparison with those who do not. The latter possibly implies that they have a rosier image of their leader, which may have led to an underrepresentation of employees who have given higher ratings for dark leadership. Future research may focus on other employees not being in educational programs yet to be better able to address generalizability of our outcomes.

### 5.2. Practical implications

Based on the results of this study, it is recommended that HR-practices are aligned with the particular context they operate in. It is important to address socio-economic and socio-cultural aspects of the context which may alter relationships that have been found, for example, in previous empirical research in other contexts. Specifically, this study shows that employees in the Netherlands and China interpret learning climate facilitation, dark leadership, employability, and turnover intention differently.

However, in both the socio-cultural national contexts studied (the Netherlands and China), our study showed that learning climate facilitation has a significant negative relationship with turnover intention. This suggests that HRM practices promoting learning climate facilitation can lower managers’ anxiety to lose employees. Especially in times of labor market shortages, the investment in learning climate facilitation may help to keep employees within the organization. This can provide organizations with a competitive advantages as they will not lose out on (firm-specific and general) knowledge and skills, or risk transferring knowledge and skills to a rival organization or potentially deterring other employees in the future.

In combination with globalization and its associated increased demands on productivity, creativity, flexibility, and accelerated developments (e.g., new production concepts and technologies), employees are required to renew their competencies across their working lives (Messmann et al., 2017; Stoffers et al., 2019, 2020). This study shows that employability and consequently dark leadership are highly relevant constructs, with highly qualified, flexible, committed employees who are focused on personal development becoming increasingly important to a firm (Atatsi et al., 2019, 2022). This new employee has lower turnover intentions through increased balance where he or she, e.g., can balance their own career goals whilst supporting their colleagues. Finally, in the Netherlands, HRM practitioners should identify dark leadership within the organization since dark leadership lowers an employee’s corporate sense even though the organization facilitates a learning climate, diminishing the positive effect of the organizational efforts.

Overall, this study showed contextual differences from the Netherlands and China, which indicates that it is important for HR-practitioners to be aware of the influence context has on the interpretation and results of HR policies and practices.

## Data availability statement

The raw data supporting the conclusions of this article will be made available by the authors, without undue reservation.

## Ethics statement

Ethical review and approval was not required for the study on human participants in accordance with the local legislation and institutional requirements. The patients/participants provided their written informed consent to participate in this study.

## Author contributions

OH, PP, BH, and JS: conceptualization. OH, PP, BH, JS, RB, and SL: methodology, writing, including the original draft’s writing, and editing. OH and SL: data curation. OH and RB: formal analysis. OH, PP, BH, JS, RB, and SL: reviewing. PP, BH, and JS: supervision. All authors contributed to the article and approved the submitted version.

## Conflict of interest

The authors declare that the research was conducted in the absence of any commercial or financial relationships that could be construed as a potential conflict of interest.

## Publisher’s note

All claims expressed in this article are solely those of the authors and do not necessarily represent those of their affiliated organizations, or those of the publisher, the editors and the reviewers. Any product that may be evaluated in this article, or claim that may be made by its manufacturer, is not guaranteed or endorsed by the publisher.
